# The diagnostic value of lower glucose consumption for *IDH1* mutated gliomas on FDG-PET

**DOI:** 10.1186/s12885-021-07797-6

**Published:** 2021-01-20

**Authors:** Feng-Min Liu, Yu-fei Gao, Yanyan Kong, Yihui Guan, Jinsen Zhang, Shuai-Hong Li, Dan Ye, Wenyu Wen, Chuantao Zuo, Wei Hua

**Affiliations:** 1 Department of Neurosurgery, Huashan Hospital, Fudan University, 12 Middle Urumqi Road, Shanghai, 200040 China; 2 Department of Neurosurgery, China-Japan Union Hospital of Jilin University, Jilin Provincial Key Laboratory of Neuro-oncology, Changchun, Jilin China; 3 PET Center, Huashan Hospital, Fudan University, Shanghai, China; 4 Department of Neurology, China-Japan Union Hospital of Jilin University, Changchun, Jilin China; 5 The Molecular and Cell Biology Lab, Institutes of Biomedical Sciences, Shanghai Medical College, Fudan University, Shanghai, China; 6 State Key Laboratory of Medical Neurobiology and MOE Frontiers Center for Brain Science, Institutes of Biomedical Sciences, Shanghai Medical College, Fudan University, Shanghai, China

**Keywords:** Glioma, Isocitrate dehydrogenase mutation, PET, Glucose, Diagnosis

## Abstract

**Background:**

Non-invasive diagnosis of IDH1 mutation for gliomas has great clinical significance, and PET has natural advantage to detect metabolism, as IDH mutated gliomas share lower glucose consumption.

**Methods:**

Clinical data of patients with gliomas and ^18^F-FDG PET were retrospectively reviewed. Receiver operating characteristic curve (ROC) analysis was conducted, and standard uptake value (SUV) was estimated in combination with grades or IDH1 mutation. The glucose consumption was investigated with U251 cells expressing wild-type or mutated IDH1 by glucose assay. Quantification of glucose was determined by HPLC in clinical tissues. Meanwhile, bioinformatics and western blot were applied to analyze the expression level of metabolic enzymes (e.g. HK1, PKM2, PC) in gliomas.

**Results:**

Seventy-one glioma cases were enrolled, including 30 carrying *IDH1* mutation. The sensitivity and specificity dependent on SUV_max_ (3.85) predicting IDH1 mutation reached 73.2 and 86.7%, respectively. The sensitivity and specificity of differentiating grades by SUVmax (3.1) were 92.3 and 64.4%, respectively. Glucose consumption of U251 IDH1 mutant cells (0.209 ± 0.0472 mg/ml) was obviously lower than IDH1wild-type cells (0.978 ± 0.0773 mg/ml, *P = 0.0001*) and astrocyte controls (0.335 ± 0.0592 mg/ml, *P = 0.0451*). Meanwhile, the glucose quantity in IDH1mutant glioma samples were significantly lower than those in IDH1 wild-type tissues (1.033 ± 1.19608 vs 6.361 ± 4.3909 mg/g, *P = 0.0051*). Silico analysis and western blot confirmed that HK1 and PKM2 in IDH1 wild-type gliomas were significantly higher than in IDH1 mutant group, while PC was significantly higher in IDH1 mutant gliomas.

**Conclusion:**

SUV_max_ on PET can predict IDH1 mutation with adequate sensitivity and specificity, as is supported by reduced glucose consumption in IDH1 mutant gliomas.

**Supplementary Information:**

The online version contains supplementary material available at 10.1186/s12885-021-07797-6.

## Background

Recent genomic investigation has been updating the molecular profiling for adult gliomas, as isocitrate dehydrogenase (IDH) mutated gliomas showed better clinical prognosis [1]. Mutated IDH induces abnormal 2-hydroxyglutarate (2-HG) accumulation, which affects the epigenetics and promotes tumorigenesis [2]. Given that IDH mutation means different treatment strategy for gliomas [3], it is important to identify IDH mutation in clinic. Post-operative immunohistochemistry staining and sequencing have been widely used to detect IDH mutation [4, 5]. Intra-operative diagnosis has being well developed by Gas chromatography-mass spectrometry (GC-MS) [6, 7], Liquid chromatography-mass spectrometry (LC-MS) [8], or antibody labelling technique [9] with adequate specificity and sensitivity.

As for pre-operative molecular diagnosis of gliomas, IDH mutation can be well recognized by identification of 2-HG on 7.0 Tesla magnetic resonance spectroscopy (MRS) [10]. Other sequences on MR, like dual phase recognition alternating gradient (DRAG)-plane echo spectrum imaging (EPSI), has also been tried to distinguish 2-HG, as 3D imaging on DRAG-EPSI produces a frequency shift 5.5 times smaller and reduce noise by 25 dB compared with conventional method [11]. It has also been reported that IDH mutation can be predicted by radiomics, or deep learning-based radiomics (DLR) from multiple modalities of MR images by convolutional neural network [12, 13]. The AUC by traditional radiomics for predicting IDH1 mutation is 86%, which is increased to 92% after DLR, and up to 95% through deep learning of multi-mode imaging data [14].

Positron emission tomography (PET) has inherent advantages on metabolic analysis, which has been used to differentiate the malignancy of brain tumors. ^18^F-fluorodeoxyglucose (FDG) is the most common PET tracer, as it is involved in the glycolysis. Other radio-labelled amino acids or ligands could also be developed for brain tumor diagnosis [15, 16]. FDG still contributed around 98% in PET examination. FDG-PET is of great significance for the diagnosis of glioma, as the textural feature of FDG-PET has showed its value on grading gliomas [17] and differentiating pseudo progression [18]. Static and dynamic FDG-PET was used to describe IDH1 genotype in gliomas [19, 20]. PET images could reveal metabolic differences among gliomas, providing a possible non-invasive preoperative molecular diagnosis with FDG, ^11^C-choline(CHO), or ^11^C-methionine(MET) [21, 22]. MET-PET/MRI has been reported to predict the IDH mutation of gliomas, by calculating the maximum tumor-brain ratio of MET uptake, and the receiver operating characteristic (ROC) analysis showed high AUC of 0.79 [23]. The biological tumor volume of early glioma differs from the standard 20–40 min FDG-PET to determine IDH status [24]. Moreover, several studies revealed an association between the FDG uptake and the status of IDH mutation or the prognosis of glioma [24, 25], as SUVmax or different ratio based on SUV showed its diagnostic value.

However, the application of PET on detecting IDH mutation is still limited in clinic. Here, we reported a simple calculation of the maximum uptake of FDG (SUV_max_) on PET showed good prediction of IDH mutation from a cohort of 71 glioma patients. ROC analysis also showed that SUV_max_ might serve as a predictor to differentiate IDH1mut vs IDH1wt gliomas, and also GBM (grade IV) vs LGG (grade II and grade III). Non-invasive and accurate prediction of IDH1 mutation in glioma has great potential in routine clinical application. Our purpose of this study was to set up a non-invasive method for prediction of IDH mutation by standard uptake value (SUV) on PET-CT with an optimal cut-off. Additionally, the consumption of glucose was reduced in IDH1mut gliomas, as demonstrated by cell line-based experiments and silico analysis. So, a simple index of FDG PET here was demonstrated to predict IDH mutation in gliomas.

## Methods

### Clinical data collection

Patients with gliomas were recruited from PET center of Huashan Hospital, Fudan University from January 2016 to December 2017. Inclusion criteria include: 1) Pathological diagnosis of gliomas, 2) Available molecular diagnosis, 3) Available pre-operative PET/CT. Totally, 71 glioma patients were enrolled, as the diagnosis of glioma (WHO II to IV) were confirmed with surgical resection and pathological examination in Department of Neurosurgery, Huashan Hospital, Fudan University. Detailed clinical information was collected and provided in the supplementary Table 1 (See detail information in supplementary Table 1). Consent forms were obtained from all patients after approval by local ethics committee. The grading of gliomas was conducted by neuropathologists according to the 2016 WHO classification of central nervous system tumors.

### Detection of IDH1 mutation

All glioma tissues obtained from the 71 patients were investigated with immunohistochemistry (IHC) using an IDH1-R132H antibody (mouse anti-human monoclonal, Dianova, German) according to the manufacturer’s instructions, and the images were reviewed by two independent neuropathologists.

### PET analysis

^18^F-FDG PET/CT was performed using integrated PET-CT scanner (Siemens Biograph 64HD PET/CT, Siemens, Germany). During the procedure, the subjects were fixed, and attenuation correction was applied with a low-dose CT (150 mAs, 120 kV, Acq. 64 × 0.6 mm). Following corrections for scatter, dead time, and random coincidences, PET images were reconstructed by 3D filtered back projection and a Gaussian Filter (FWHM 3.5 mm), providing 64 continuous trans-axial slices of 5-mm-thick spacing. PET-CT images were interpreted by experienced radiologists. The FDG uptake was normalized using the standardized uptake value (SUV) by dividing the radioactivity (kBq/mL) in the brain by the radioactivity injected per gram of body weight. The background area is defined in the symmetrical cerebellar cortex. SUVmax was used as the reference measurement and was determined by considering the tumor uptake given by the maximum pixel value within the gliomas [26].

### Cell culture

Human glioma cell lines normal astrocytes (NHA, gift from Shandong university), U251 (purchased from Genechem, GCC-GL0001RT,2017, provided in the Supplementary 3), supplemented with 10% fetal bovine serum (Meilunbio) in culture medium and stored at 37 °C in 5% carbon dioxide humidification atmosphere. When the cell density reaches 80% ~ 90%, remove the medium and wash with 10 ml PBS twice. Trypsin digestion with 0.25% EDTA was added, centrifuged, inoculated as required, and cultured in a cell culture tank containing 5% CO_2_. Meanwhile, the cell lines (U251 IDH1mut, U251 IDH1wt provided in the Supplementary 3) were established in the lab of Institute of Biomedical Sciences, Shanghai Medical College, Fudan University, Shanghai, China [27].

### Glucose assay

Cells in logarithmic growth phase with good growth status were taken and inoculated into 6-well plates with 4 × 10^5^ cells per well. 3–4 multiple Wells were set in each group. After continuous culture for 1 Day, cell glucose consumption was detected, according to the kit operation method (GAHK20, Glucose (HK) Assay Kit).

### Glucose quantitation with HPLC

Another batch of untreated glioma tissue were collected for HPLC analysis (See detail information in supplementary Table 2). From the liquid nitrogen frozen storage samples (IDH1mut, IDH1wt and normal tissue), weigh 50 mg of each sample, cut up and put samples into 1.5 ml EP tube, then add 1 ml 80°Cprecooling methanol-water mixture solution (4:1, v/v), add D-GLUCOSE-13C_6_ as internal standard, blending for 10 s, and then grinding 3 min in 60 HZ frequency in the grinding apparatus, After the sample is grounded, the EP tube is placed at − 80°Cfor 12 h. Centrifuge at 4 °C, rotate at 12000 r/min for 10 min, then collect the supernatant into a 1.5 ml EP tube and centrifuge in a vacuum concentrator. The dried extract was dissolved in 50ul phase A. After blending, centrifuge at a speed of 12,000 r/min for 10 min. Transfer the supernatant into a disposable syringe and connect it with a 0.22um filter column for filtration. The filtrate was transferred into a special injection bottle for High Performance Liquid Chromatography (HPLC) analysis (HPLC-QQQ-MS, Amino column type: Phenomenex P/N 00b-4378-b07 Luna NH2 [50*2.0 mm] with pore diameter of 5um and column temperature maintained at 40 °C).

### Western blot

Three cases of normal tissue, 8 cases of IDH1mut and 6 cases of IDH1wt gliomas were used for western blot (See detail information in supplementary Table 3). To analyze the expression of Hexokinase1(HK1), Pyruvate Carboxylase (PC), Pyruvate KinaseM2(PKM2), Western blot assays were performed using the following primary antibodies: rabbit anti-human HK1 (C35C4, Cell Signalling Technology; 1:1000), PC(ab12607, abcam; 1:1000), PKM2(D78A4, CellSignallingTechnology;1:1000) and Rabbit anti-actin (arigobio; 1:5000). Briefly, tissues were lysed with RIPA buffer (50 mM Tris– HCl [pH 7.4], 150 mM NaCl, 1% Triton X-100, 1% sodium deoxycholate,0.1% SDS, Sodium orthovanadate, EDTA and many other inhibitors) containing protease inhibitors (Roche, Complete Mini); 20 μg samples of the lysates were separated on 10% SDS-PAGE gels and transferred to NC membranes. The membranes were incubated with primary antibodies overnight at 4 °C. The primary antibody incubation was followed by incubation with an HRP-conjugated secondary antibody. The bound antibodies were detected using an ECL kit (Thermo, Prod#34095).

### Bioinformatic analysis

TCGA Dataset of glioma was downloaded from TCGA data portal (http://tcgadata.nci.nih.gov). We extracted gene expression data using the “ data matrix “ tool provided by TCGA data porta. Related genes including Hexokinase (HK), 6-phosphofructokinase-1 (FPK1), Pyruvate kinase (PK), Fructose diphosphatase 1 (FBP1), Glucose- 6phosphatase (G6PC), Phosphoenolpyruvate carboxyl kinase (PEPCK), Pyruvate Carboxylase (PC), were selected for analysis among IDH1mut and IDH1wt gliomas. Significant genes (*P* < 0.05) were shown with a scatter plot.

### Statistical analysis

In order to evaluate the diagnostic accuracy of FDG-PET SUV_max_ for IDH1 genotype prediction, the collected data were divided into two groups according to IDH1wt and IDH1mut. Then the samples were further divided into lower grade gliomas (grade II and grade III, LGG) and glioblastoma (grade IV, GBM). IDH1 status or grades were taken as reference and the diagnostic accuracy was evaluated by analyzing the ROC curve. When the diagnostic accuracy reaches the maximum, the decision cutoff is considered to be optimal.

## Results

### IDH1-mutated gliomas showed lower glucose uptake on PET

**Fig. 1:**
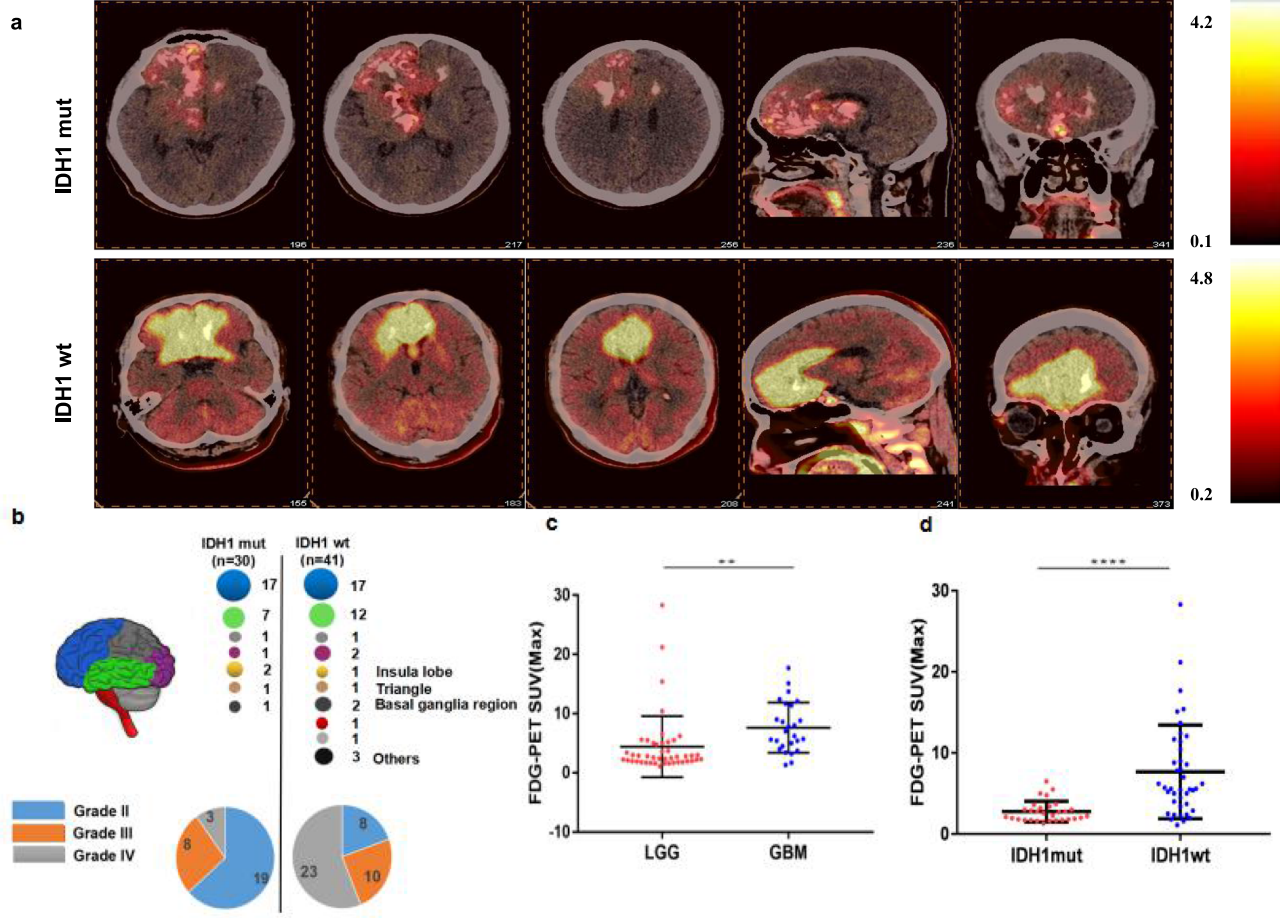
Typical PET-CT images and analysis. Typical FDG-PET images of IDH1mut (upper panel) and IDHwt (lower panel) gliomas are shown (**a**). Clinical characteristics of the patients are illustrated, as the circle represents sample size, and the color corresponds to the tumor location. The ratio of IDH1mut in LGG is significantly higher than in GBM (**b**). The higher grade of gliomas exhibits higher value of glucose uptake (**c**). SUVmax of glucose uptake in IDH1mut gliomas on analysis is significantly lower than in IDH1wt gliomas (**d**). *P < 0.05, **P < 0.01, ***P < 0.001

Seventy-one patients (44 M and 27F, aged from 12 to 73 years old) with gliomas were enrolled, including 45 patients diagnosed with LGG (27 grade II and 18 grade III) and 26 patients diagnosed with GBM (26 grade IV). 30 cases harbored IDH1mut (19 grade II, 8 grade III and 3 grade IV), and the other 41 cases were IDH1wt (8 grade II, 10 grade III and 23 grade IV). Among these gliomas (detail information in Fig. 1b), the ratio of IDH1mut in LGG (90%) was significantly higher than in GBM (10%). SUVmax of FDG uptake in LGG (4.407 ± 5.172) was lower than in GBM (7.632 ± 4.243) (*P* = 0.0088, Fig. 1c). SUV_max_ of FDG uptake in IDH1mut gliomas was around 2.761 ± 1.275 on FDG-PET analysis, which is significantly lower than 7.656 ± 5.780 in IDH1wt gliomas (*P<0.0001*, Fig. 1d). There is a significant difference of SUVmax between IDHwt and IDHmt gliomas Our data showed that there is no difference in SUVmax between astro- vs oligo-type LGG (supplementary Fig. 1). Typical images of FDG uptake of different types of gliomas on PET-CT were shown in Fig. 1a.

### SUV_max_ of FDG uptake on PET differentiates IDH1 mutation and grades

**Fig. 2:**
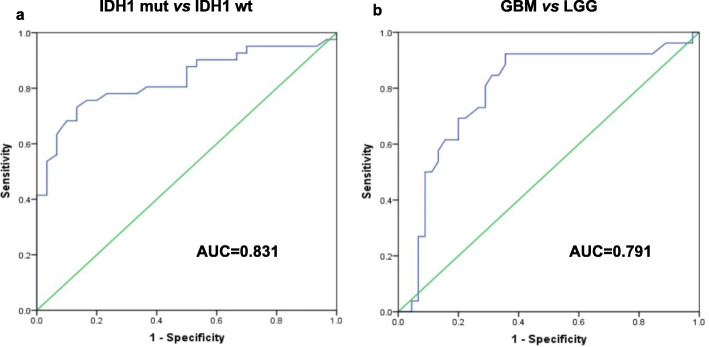
SUVmax on PET could differentiate IDH1 mutation and grades. ROC analysis shows that SUVmax of 3.85 may serve as a threshold for predicting IDH1mut and IDH1wt (**a**), and that SUVmax of 3.1 can serve as a predictor to differentiate GBM and LGG (**b**)

ROC analysis suggested that SUV_max_ of 3.85 could serve as a threshold for predicting IDH1mutation (AUC = 0.831, 95%CI 0.736 to 0.927, *P<0.0001*), and the sensitivity of differentiating IDH1mut and IDH1wt gliomas reaches 73.2%, while the specificity reaches 86.7% (Fig. 2a). ROC analysis also showed that SUV_max_ of 3.1 could serve as a predictor to differentiate GBM vs LGG (AUC = 0.791, 95%CI 0.675 to 0.906, *P<0.0001*), as the sensitivity and specificity of differentiating GBM and LGG reaches 92.3 and 64.4%, respectively (Fig. 2b).

### IDH1 mutation inhibits the glucose consumption in gliomas

**Fig. 3:**
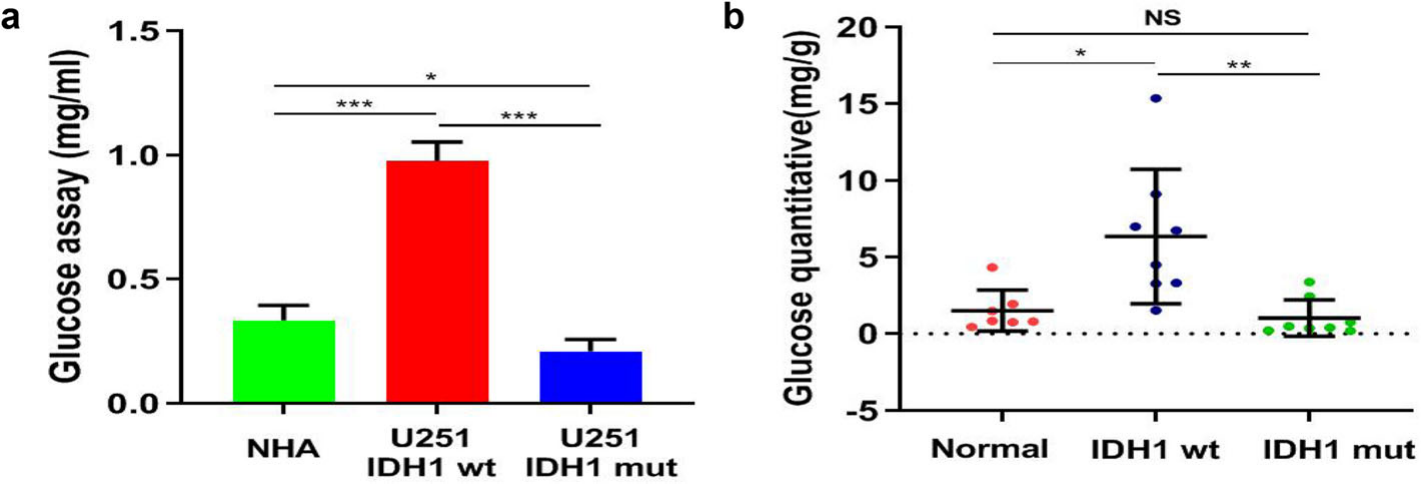
Glucose consumption and quantitative assay. The glucose consumption of U251 cells expressing IDH1mut is lower than cells expressing IDH1wt and NHA control cells (**a**). The glucose quantity in IDH1wt tissues is significantly higher than that of IDH1mut gliomas (**b**)

The effect of IDH1 mutation on glucose uptake were investigated with U251 expressing IDH1wt or IDH1mut as well as normal astrocytes lines (NHA). We found that the glucose consumption in IDH1mut cells was obviously lower than cells expressing IDH1wt (0.209 ± 0.0472 mg/ml vs 0.978 ± 0.0773 mg/ml, *P = 0.0001*) and NHA controls (0.335 ± 0.0592 mg/ml, *P = 0.0451*, Fig. 3a). The quantitation of glucose by HPLC in clinical glioma samples (8 IDH1mut, 8 IDH1wt, and 7 normal brain) were also analyzed, and the results showed that the level of glucose in IDH1wt samples were significantly higher than those in IDH1mut ones (6.361 ± 4.3909 mg/g vs 1.033 ± 1.19608 mg/g, *P = 0.0051*, Fig. 3b**)**. Taken together, these findings suggest that glucose consumption is decreased in both IDH1mut cell line and clinical samples, which is in accordance with the lower FDG uptake on PET.

### IDH1 mutation affects the expression of enzymes for carbohydrate metabolism

**Fig. 4:**
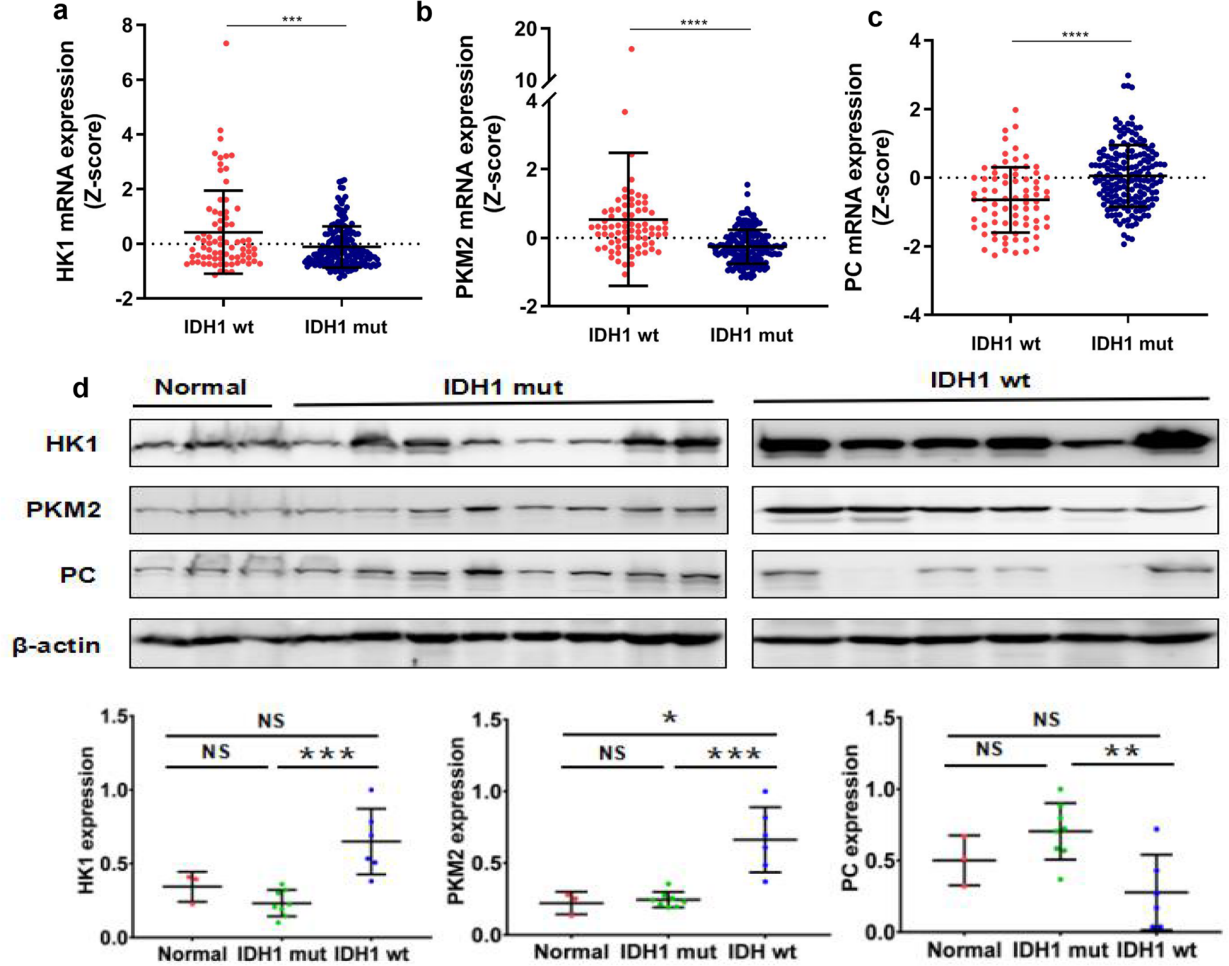
Expression of HK1, PKM2, and PC in gliomas. Bioinformatic analysis of TCGA data shows that the mRNA of HK1 and PKM2 is higher in IDH1wt than that in IDH1mut gliomas (**a**, **b**), while PC expression is significantly higher in IDH1mut than that in IDH1wt gliomas (**c**). Furthermore, the up regulation of HK1 and PKM2 in IDH1wt gliomas are confirmed with western blot, while the expression of PC is higher in IDH1mut gliomas than that in IDH1wt group, the gels/blots were processed in parallel (**d**, full-length blots/gels are presented in Supplementary 2 Fig. [X])

The expression of enzymes involved in the carbohydrate metabolism, including Hexokinase (HK), 6-phosphofructokinase-1 (FPK1), Pyruvate kinase (PK), Fructose diphosphatase 1 (FBP1), Glucose- 6phosphatase (G6PC), Phosphoenolpyruvate carboxyl kinase (PEPCK), Pyruvate Carboxylase (PC), were analyzed with TCGA datasets. Silico analysis showed that HK1 and PKM2 in IDH1wt gliomas were significantly higher than those in IDH1mut group (*P = 0.0002* and *P<0.0001*, respectively), while PC was significantly higher in IDH1mut than in IDH1wt gliomas (*P<0.0001*, Fig. 4a-c**)**. Western blot also confirmed the up-regulation of HK1 and PKM2 in IDH1wt gliomas (*P = 0.0004* and *P = 0.0003*, respectively). Instead, the expression of PC was higher in IDH1mut gliomas than in IDH1wt group (*P = 0.0046*). No difference of PC expression was observed between IDH1mut gliomas and normal brain tissues (*P* = 0.1536, Fig. 4d**)**. These findings indicate that the glycolysis is inhibited, while glycogenesis is stimulated by IDH1 mutation, which is in line with reduced glucose consumption in IDH1mut gliomas.

## Discussion

Previous studies suggested that IDH1 mutation is a powerful prognostic marker for gliomas [28], and it is also considered as a predictive biomarker for extensive surgical resection, radiotherapy and chemotherapy in gliomas [29]. So, IDH1 mutation has been applied in molecular typing and comprehensive diagnosis for gliomas [30, 31]. In this study, preoperative molecular profiling of IDH1mut was conducted with PET analysis, and SUV_max_ of glucose uptake of 3.85 could differentiate IDH1mutation with sensitivity of 73.2% and specificity of 86.7%. In the meantime, SUV_max_ of 3.1 could distinguish LGG from GBM with sensitivity of 92.3% and specificity of 64.4%. This FDG-PET analysis might provide a simple reference for preoperative molecular subtyping of gliomas.

FDG-PET could help to map areas of rapid proliferative tumors for its nature advantage on metabolic detection [32]. The ratio of FDG uptake in tumor to white matter (cutoff = 1.5) has been used for identifying low and high grade gliomas on PET, and early changes of FDG uptake have been reported as a prognostic factor [33]. Many methods were reported that can be used for preoperative prediction of IDH1 genotype by using MRS [34] or structural feature analysis on MR [35], as the accuracy with preoperative MR could reach 89% [36]. Deep learn-based radiomics could raise the accuracy to 85–95% [14]. However, this sophisticated method still needs training and further validation with big cohort, which might contradict the non-invasive advantage during clinical management. The texture characteristics combined with static and dynamic parameters of electrostatic field effect transistor absorption were used in noninvasive prediction of IDH1 genotype by O-(2-[^18^F] fluoroethyl)-L-tyrosine) FET-PET radiomics, and the accuracy of diagnosis was significantly improved. In addition, the combination of standard PET parameters with texture features could also significantly improve the accuracy. The highest diagnostic accuracy of the PET/MR hybrid scanner was 93% in predicting IDH1 genotypes [37]. SUV_max_ with the optimal cut-off was calculated through all patients in this study, which is simple and easy in clinic use, although the accuracy still needs to be further improved. SUV_max_ here has been normalized to the brain and background, as our radiologists had differentiate disease-related changes from age-related changes in brain metabolism [36].

FDG-PET could predict IDH1 mutation non-invasively and pre-operatively, and it could also identify LGG and GBM hereby. With the update of molecular subtyping for gliomas, it is necessary to re-evaluate previous studies. Grade II and III gliomas share similar molecular features as lower grade, instead of low- and high-grade gliomas. Our results validated this subtyping with PET analysis, and its nature metabolic advantage is good enough to identify LGG and GBM. The ratio of IDH1 mutation in LGG is 80% [38], and it is as low as 12% in GBM [39]. In Chinese cohort, the ratio could be different [40], as IDH1 mutation in GBM is high here. Since IDH1 mutation have a great impact on glucose metabolism [41], which is also supported by cell line and bioinformatics analysis. IDH1 mutation might contribute to the lower glucose metabolism and good prediction of malignancy in gliomas. Kim D et al. analyzed a cohort o 59 gliomas and found that the ratio of SUVmax of glioma to SUVmean of the contralateral cortex (G/C ratio) was correlated with IDH1 mutation [25], which also supported our conclusion. Li L et al. tried to combine PET and radiomics to predict IDH mutation and prognosis [21].

IDH1mut and IDH1wt cells could be easily differentiated by metabolic expression profiles and glucose consumption [42]. In order to clarify the influence of IDH1 mutation on glucose consumption and the level of glucose in gliomas, we compared the expression of key gluconeogenesis and glycolysis enzymes in clinical tissues. With expectation, IDH1 mutation could inhibit HK1 and PKM2. Both bioinformatics analysis and clinical validation have shown that IDH1 mutation could significantly alter gluconeogenesis and glycolysis, which contributes to the lower glucose consumption in IDH1mut gliomas. Chen et al found that cell growth was significantly inhibited either by IDH1 knockout or mutant, which could be restored after the reintroduction of IDH1, and glucose transport is the key pivot during this event [41]. IDH1 mutation could dramatically affect tumor metabolism and epigenetics, as proline is upregulated simultaneously with 2-HG in IDH1mut gliomas in our previous study [43]. Glutamate dehydrogenase 2 (GLUD2) also could compensate to synthesize α-KG in IDH1mut gliomas, thereby resisting the growth inhibition caused by IDH1 mutation. So, there might be multiple manners that IDH1 mutation could affect glucose consumption.

## Conclusions

In conclusion, SUV_max_ on FDG-PET showed adequate sensitivity and specificity in predicting IDH1 mutation. In this study, the sensitivity and specificity were adequate, which may be due to sample size and other metabolic mechanism. IDH2 mutation contribute a small population of gliomas, which cannot be identified by PET analysis now. The combination with other parameters or MR, or DLR definitely could improve the accuracy. Here, a simple way of SUV_max_ was tried to predict IDH1 mutation and grades, which seems quite feasible for clinicians. With the development of PET, more accurate method will be used for better diagnosis of gliomas in the future.

## Supplementary Information

**Additional file 1 Supplementary Table 1**: Characteristic of patient population subjected to pathological evaluation, related to Fig. 1.

**Additional file 2.**

**Additional file 3.**

**Additional file 4.**

**Additional file 5 Supplementary Fig. 1.** Our data showed a significant FDG SUVmax differences between grade IV gliomas of IDHwt and IDHmt, Our data showed that there is no difference in FDG SUVmax between astro- vs oligo-type lower grade gliomas, related to Fig. 1.

**Additional file 6.**

## Data Availability

The datasets used and/or analyzed during the current study are available from the corresponding author on reasonable request.

## Abbreviations

ROC: Receiver operating characteristic
^18^F-FDG: 18-F-fluorodeoxyglucose
PET: Positron emission tomography
SUV: Standard uptake value
IDH1: Isocitrate dehydrogenase1
WHO: World Health Organization
MRS: Magnetic resonance spectra
2-HG: 2-hydroxyglutarate
HK1: Hexokinase 1
PKM2: Pyruvate Kinase M2
PC: Pyruvate Carboxylase
GBM: Glioblastoma, grade IV
LGG: Lower grade gliomas, grade II and grade III
