# Supplementary material for: The diagnostic value of lower glucose consumption for IDH1 mutated gliomas on FDG-PET
Source: BMC Cancer. 2021 Jan 20;21:83. doi: 10.1186/s12885-021-07797-6 (PMC7816361; doi:10.1186/s12885-021-07797-6)
Supplement: Supplementary file 1 — Additional file 1 Supplementary Table 1: Characteristic of patient population subjected to pathological evaluation, related to Fig. 1. [file 12885_2021_7797_MOESM1_ESM.docx]

**Supplementary Table 1:** Characteristic of patient population subjected to pathological

evaluation, related to Figure 1.

|  | *IDH1* Mutation  (n=30) | *IDH1* Wild-type  (n=41) | P value |
| --- | --- | --- | --- |
| Age (yr)  Range(median) | 25-69(44) | 12-73(49) | P=0.1693 |
| Sex  Male  Female | 18(60%)  12(40%) | 26(63.4%)    15(36.6%) | P=0.8871  P=0.0256 |
| Grade  II  III  IV | 18(60%)  9(30%)  3(10%) | 9(22%)  9(22%)  23(56%) | P=0.8564  P=0.0247  P=0.0124 |
| Tumor type  Diffuse astrocytoma  Oligoastrocytoma  Oligodendroglioma  Anaplastic astrocytoma  astrocytoma  Anaplastic oligodendroglioma  GBM | 4(13.3%)  1(3.3%)  10(33.3%)  5(16.7%)  3(10%)  4(13.4%)  3(10%) | 2(4.9%)  0(0%)  2(4.9%)  9(22%)  5(12.2%)  0(0%)  23(56%) |  |
| Location  Corpus callosum  Insular lobe  Parietal lobe  Frontal lobe  Temporal lobe  Occipital lobe  Brain stem, Thalamus  Pineal gland  Triangle  Cerebellum  Basal ganglia | 0(0%)  2(6.7%)  1(3.3%)  17(56.7%)  7(23.4%)  1(3.3%)  0(0%)  0(0%)  1(3.3%)  0(0%)  1(3.3%) | 2(4.9%)  1(2.4%)  1(2.4%)  17(41.5%)  12(29.4%)  2(4.9%)  1(2.4%)  1(2.4%)  1(2.4%)  1(2.4%)  2(4.9%) |  |

GBM, glioblastoma multiforme.
