# Supplementary material for: The diagnostic value of lower glucose consumption for IDH1 mutated gliomas on FDG-PET
Source: BMC Cancer. 2021 Jan 20;21:83. doi: 10.1186/s12885-021-07797-6 (PMC7816361; doi:10.1186/s12885-021-07797-6)
Supplement: Supplementary file 2 — Additional file 2. [file 12885_2021_7797_MOESM2_ESM.doc]

U251 (purchased from Genechem, GCC-GL0001RT, 2017); the cell lines (U251 IDH1mut, U251 IDH1wt ) were established in the lab of Institute of Biomedical Sciences, Shanghai Medical College, Fudan University, Shanghai, China .the cell line has recently been authenticated (Cell Line U251 STR Profile Report_1417).This cell line is recently free of mycoplasma contamination.The cell lines are purchased directly through the company and have been ethically approved.
