# Supplementary material for: The diagnostic value of lower glucose consumption for IDH1 mutated gliomas on FDG-PET
Source: BMC Cancer. 2021 Jan 20;21:83. doi: 10.1186/s12885-021-07797-6 (PMC7816361; doi:10.1186/s12885-021-07797-6)
Supplement: Supplementary file 3 — Additional file 3. [file 12885_2021_7797_MOESM3_ESM.docx]

| Number | Age | Diagnosis | Grade | Location | IDH1 |
| --- | --- | --- | --- | --- | --- |
| Patient 1 | 30-35 | astrocytoma | II | The left temporal lobe | (+) |
| Patient 2 | 35-40 | astrocytoma | II | Left temporal occipital lobe | (+) |
| Patient 3 | 30-35 | astrocytoma | II | Right frontal lobe | (+) |
| Patient 4 | 45-50 | astrocytoma | II | The left frontal lobe | (+) |
| Patient 5 | 30-35 | Oligoastrocytoma | II | Right frontal lobe | (-) |
| Patient 6 | 50-55 | astrocytoma | II | The left frontal lobe | (+) |
| Patient 7 | 25-30 | astrocytoma | II | The left frontal lobe | (-) |
| Patient 8 | 65-70 | Anaplastic astrocytoma | III | The right temporal lobe | (-) |
| Patient 9 | 40-45 | Anaplastic astrocytoma | III | The right temporal lobe | (-) |
| Patient 10 | 50-55 | Anaplastic oligodendroglioma | III | Right frontal lobe | (+) |
| Patient 11 | 45-50 | Anaplastic astrocytoma | III | Right frontal parietal lobe | (-) |
| Patient 12 | 45-50 | Anaplastic oligodendroglioma | III | Right frontal lobe | (+) |
| Patient 13 | 60-65 | Anaplastic oligodendroglioma | III | The left frontal lobe | (+) |
| Patient 14 | 60-65 | GBM | IV | The left temporal lobe | (-) |
| Patient 15 | 35-40 | GBM | IV | Right basal ganglia | (-) |
| Patient 16 | 65-70 | GBM | IV | The right parietal lobe | (-) |
| Patient 2 | 35-40 | Normal |  |  |  |
| Patient4 | 45-50 | Normal |  |  |  |
| Patient5 | 30-35 | Normal |  |  |  |
| Patient8 | 65-70 | Normal |  |  |  |
| Patient6 | 50-55 | Normal |  |  |  |
| Patient12 | 45-50 | Normal |  |  |  |
| Patient13 | 60-65 | Normal |  |  |  |
