# Supplementary material for: The diagnostic value of lower glucose consumption for IDH1 mutated gliomas on FDG-PET
Source: BMC Cancer. 2021 Jan 20;21:83. doi: 10.1186/s12885-021-07797-6 (PMC7816361; doi:10.1186/s12885-021-07797-6)
Supplement: Supplementary file 4 — Additional file 4. [file 12885_2021_7797_MOESM4_ESM.doc]

| Number | Age | Diagnosis | Grade | Location | IDH1 |
| --- | --- | --- | --- | --- | --- |
| Patient 17 | 35-40 | Oligoastrocytoma | II | The left frontal lobe | (+) |
| Patient18 | 40-45 | astrocytoma | II | Right frontal lobe | (+) |
| Patient19 | 35-40 | astrocytoma | II | The left frontal lobe | (+) |
| Patient20 | 50-55 | GBM | IV | The left frontal lobe | (+) |
| Patient21 | 40-45 | astrocytoma | II | Right frontal lobe | (+) |
| Patient22 | 45-50 | Anaplastic oligodendroglioma | III | The left frontal lobe | (+) |
| Patient23 | 45-50 | astrocytoma | II | Right frontal parietal lobe | (+) |
| Patient24 | 50-55 | astrocytoma | II | The left frontal lobe | (+) |
| Patient25 | 65-70 | GBM | IV | The left parietal lobe | (-) |
| Patient26 | 60-65 | Anaplastic astrocytoma | III | Right frontal temporal lobe | (-) |
| Patient27 | 60-65 | GBM | IV | The right parietal lobe | (-) |
| Patient28 | 45-50 | GBM | IV | The left parietal lobe | (-) |
| Patient29 | 70-75 | Recurrent GBM | IV | Right frontal lobe | (-) |
| Patient30 | 70-75 | GBM | IV | The left temporal lobe | (-) |
| Patient20 | 50-55 | Normal |  |  |  |
| Patient26 | 60-65 | Normal |  |  |  |
| Patient18 | 40-45 | Normal |  |  |  |
