# Supplementary material for: The diagnostic value of lower glucose consumption for IDH1 mutated gliomas on FDG-PET
Source: BMC Cancer. 2021 Jan 20;21:83. doi: 10.1186/s12885-021-07797-6 (PMC7816361; doi:10.1186/s12885-021-07797-6)
Supplement: Supplementary file 6 — Additional file 6. [file 12885_2021_7797_MOESM6_ESM.doc]

**All the pictures are original, not cropped**







**Normal**

IDH1 mut

IDH1 wt

100

100

**HK1**

75

75

supplementary figure 4d (HK1)

**



**

63

48

IDH1 mut

IDH1 wt

48

63

**PKM2**

**Normal**

supplementary figure 4d (PKM2)







48

48

**PC**

**Normal**

IDH1 mut

IDH1 wt

35

35

supplementary figure 4d (PC)







48

63

48

63

**Normal**

IDH1 mut

IDH1 wt

35

35

48

**β-actin**

supplementary figure 4d (β-actin)
