# Supplementary material for: The diagnostic value of lower glucose consumption for IDH1 mutated gliomas on FDG-PET
Source: BMC Cancer. 2021 Jan 20;21:83. doi: 10.1186/s12885-021-07797-6 (PMC7816361; doi:10.1186/s12885-021-07797-6)
Supplement: Supplementary file 5 — Additional file 5 Supplementary Fig. 1. Our data showed a significant FDG SUVmax differences between grade IV gliomas of IDHwt and IDHmt, Our data showed that there is no difference in FDG SUVmax between astro- vs oligo-type lower grade gliomas, related to Fig. 1. [file 12885_2021_7797_MOESM5_ESM.zip › Supplementary Figures and legendsR6.doc]

**Supplementary Figure 1.**our data showed a significant FDG SUVmax differences between grade IV gliomas of IDHwt and IDHmt, Our data showed that there is no difference in FDG SUVmax between astro- vs oligo-type lower grade gliomas, related to Figure 1.
